# Polymorphisms of Encoding Genes *IL1RN* and *P2RX7* in Apical Root Resorption in Patients after Orthodontic Treatment

**DOI:** 10.3390/ijms22020777

**Published:** 2021-01-14

**Authors:** Agata Ciurla, Jolanta Szymańska, Bartosz J. Płachno, Anna Bogucka-Kocka

**Affiliations:** 1 Dentist’s Office ORTO-PUNKT, Mościckiego St. 72/1, 33-100 Tarnów, Poland; ortopunkt.tarnow@gmail.com; 2 Department of Integrated Pediatric Dentistry, Chair of Integrated Dentistry, Medical University of Lublin, 20-059 Lublin, Poland; 3 Department of Plant Cytology and Embryology, Institute of Botany, Faculty of Biology, Jagiellonian University in Kraków, 9 Gronostajowa St., 30-387 Cracow, Poland; bartosz.plachno@uj.edu.pl; 4 Department of Biology and Genetics, Medical University of Lublin, 4a Chodźki St., 20-093 Lublin, Poland; anna.bogucka-kocka@umlub.pl

**Keywords:** gene polymorphism, orthodontic treatment, root resorption

## Abstract

External apical root resorption (EARR) is one of the most serious complications associated with orthodontic treatment. The aim of the study was to analyze the relationships between selected single nucleotide polymorphisms (SNPs) in Interleukin 1 receptor antagonist (*IL1RN*), purinoreceptor P2X7 (*P2RX7*) and EARR in patients after orthodontic treatment. The study comprised 101 patients who underwent a complex orthodontic treatment with a combination of fixed appliances. Roots were measured based on orthopantomograms and lateral cephalometric radiographs taken before and at the end of the treatment using diagnostic software. Proportional measurements of selected teeth were made using the modified Linge and Linge methods. Based on the presence or absence of EARR, patients were divided into two groups: control group, 61 patients without EARR (with 0.90 ≤ rRCR ≤ 1.00), and EARR group, 40 patients with EARR (rRCR < 0.90). Root resorption in selected groups was also evaluated with the scores of Malmgren and Levander. SNP analysis was performed using the real-time polymerase chain reaction (PCR) method. The analysis indicated that a specific haplotype of *P2RX7* (rs208294) and *IL1RN* (rs419598) modified the risk of EARR development (*p* < 0.05), with a Bonferroni correction. The analysis of the *P2RX7* and *IL1RN* gene polymorphisms showed that the presence of SNPs of these genes may predispose individuals to EARR. These findings indicate that EARR is a complex condition influenced not only by environmental factors and needs further study on the genetic risk factors.

## 1. Introduction

External apical root resorption (EARR) is one of the most serious complications associated with orthodontic treatment [1,2,3]. Before the start of the orthodontic treatment patients are informed about all the complications that may occur during or after the treatment. Dynamic development in the field of orthodontics gives the patients a wider choice of aesthetic appliances, shorter treatment time, reduction of the need for cooperation and almost guarantees the success of the therapy. Achieving an aesthetic result and correct occlusal conditions is not synonymous with achieving therapeutic success. An important element of the results of orthodontic treatment is the analysis of panoramic images taken before and after or just before the end of the active treatment phase [4,5]. The roots should not be in contact, long root axis must be parallel and the shape of the roots should not differ from the shape prior to treatment. A change in the root shape, such as apical rounding, slightly blunted or round apex to a grossly resorbed apex, indicates a resorption process [6].

In 2–5% of cases, this may lead to a significant root length loss (>5 mm) [3,7,8]. When resorption occurs, therapeutic success is questionable despite the ideal occlusion and the patient’s satisfaction with a beautiful smile.

Root resorption, as a side effect of orthodontic treatment, has been known for a century [9]. In primary dentition it is a physiological phenomenon. As an undesirable pathological process in permanent teeth, it can occur even in orthodontically untreated people (7–13%), but it more often concerns the roots of teeth exposed to orthodontic forces [10,11]. In people affected by resorption, who do not wear orthodontic appliances, EARR is presumably a reaction to the excessive occlusive forces such as parafunctional habits [10,12]. Brezniak and Wasserstein proposed the division of factors causing pathological resorption into biological, mechanical, combined biological and mechanical products, and others [13]. Mechanical factors initiate the process of root resorption as a result of a local inhibition of blood circulation under pressure. Biological factors are responsible for the development of resorption, affecting its progress, as well as for the control of physiological and morphological changes in the periodontium. Vlascalic et al. divided the main causes of EARR into two groups: factors related to the patient, such as genetic predisposition, age, gender, race, group of resorptive teeth, root shape of teeth, and factors related to orthodontic treatment, e.g., the type of malocclusion, type of orthodontic appliance, duration of treatment or excessive orthodontic forces [14]. Since 1975, when Newman announced that EARR includes family occurrence, EARR has become associated with genetics [15]. During hereditary risk studies, Hartsfield et al. focused on the genes encoding the proteins involved in bone remodeling and modeling [3]. Other studies have mentioned the significant role of proteins involved in the modulation of the inflammatory response (ATP/P2RX7/IL-1b) and regulatory proteins of osteoclast activation (RANK/RANKL/OPG) [16,17,18].

The *P2RX7* is expressed by osteoblasts and osteoclasts and activates osteoblast function and apoptosis [19,20]. It is also involved in the inflammatory process by stimulating the release of cytokines such as IL-1b [21]. Hartsfield suggested two pathways that play role in the development of inflammatory EARR [22]. The ATP/P2RX7/IL-1b inflammation pathway is inducing osteoclasts [20,23] and the RANK/RANKL/OPG system is involved in the bone and root remodeling [24].

Interleukin 1 is also involved in the inflammatory process [25] by amplifying osteoclast differentiation, which at the end of the cascade process leads to root resorption [26]. Iwasaki and coworkers found higher concentration of IL1b in crevicular fluid and gingival tissues during orthodontic treatment [27]. *IL1RN* gene is encoding one of three molecules in the IL1 cluster, IL1 receptor antagonist (IL1Ra). Therefore, genes involved in remodeling and inflammatory process were chosen to evaluate genetic susceptibility to EARR.

The *P2RX7* gene is located on chromosome 12 of humans. The polymorphism within this gene is the transition or transversion of T > C/A/G in position 121162450. In the polymorphism studied, a change of nitrogen bases within a given group or between groups is observed: conversion of T-thymine to C-cytosine, A-adenine or G-guanine. The TAT codon encoding tyrosine (Tyr) exchanges T > C. As a result of the mutation, the amino acid is replaced, tyrosine (Tyr) is replaced by histidine (His). If the thymine replaces adenine, the AAT codon encoding asparagine is formed and the conversion of thymine with guanine results in the formation of aspartic acid (GAT).

The *IL1RN* gene is located on human chromosome 2. The polymorphism within this gene consists in the T > C transition at position 113129630. In the polymorphism studied (rs419598), a change of nitrogen bases within pyrimidine bases is observed: thymine exchange for cytosine. The majority of the population has T. This polymorphism does not affect the change in protein because the conversion of T to C in the GCT codon results in the creation of a GCC codon, which, similarly to the GCT codon, codes for alanine (Ala).

The influence exerted by EARR by polymorphisms in the selected genes have been analyzed in different samples. Linhartova et al. in their study searched for possible connections between selected treatment-related clinical parameters and single nucleotide polymorphisms (SNPs) in the genes *P2RX7*, IL-17A, SPP1, TNFRSF11B and EARR in the Czech population undergoing orthodontic treatment [28]. The effect of SNP *P2RX7*, IL-17, SPP1 or TNFRSF11B on the development of EARR has not been confirmed in the Czech population. However, it has been shown that *P2RX7* gene variation can be a causal factor of postorthodontic EARR. Iglesias-Linares et al. showed that the TT allele of SNP in the IL-1RN gene (rs419598) was strongly and positively correlated with EARR [29]. It is typical that in multifactorial diseases, such as EARR, each genetic polymorphism has only a partial influence on the clinical phenotype, which is also modified by environmental factors.

The aim of the study was to analyze the possible relationships between the occurrence of external resorption in the maxillary and mandibular incisors and the first molars of the upper jaw and variability in the *P2RX7* (+489C/T, rs208294) and *IL1RN* (+2018T/C, rs419598) genes in patients treated with fixed appliances.

## 2. Results

The conducted studies prove that in the majority of patients conducting orthodontic treatment, no serious root resorptions occurred. Shortening of roots below 90% of their length concerned 9.21% of all the examined teeth, of which the advanced apical root resorption with the loss over 20% of root length (rRCR < 0.80) occurred in 23 out of 1010 analyzed teeth (2.28%) (Table 1).

Among the study group, in 5.75% of the examined teeth, advanced apical root resorption with the loss above 20% of root length occurred, while 17.25% of teeth were shortened by 10–20% of their initial length (Table 2).

In patients from the control group, in the panoramic photographs taken at the end of the treatment, neither advanced nor moderate resorptions with the loss above 10% of root length were found. Mild apical root resorption was found in 98.03% of the examined teeth, while in 12 teeth (1.97%) no signs of resorption were found (Table 3).

Advanced apical root resorption leading to roots shortening by over 80% of their initial length mainly concerned maxillary right lateral incisors (4.98% of all teeth) and mandibular left central incisors (3.96% of all teeth) (Table 4). Upper first molars were not subjected to advanced resorption with the loss of over 20% of roots length.

**Figure 1:**
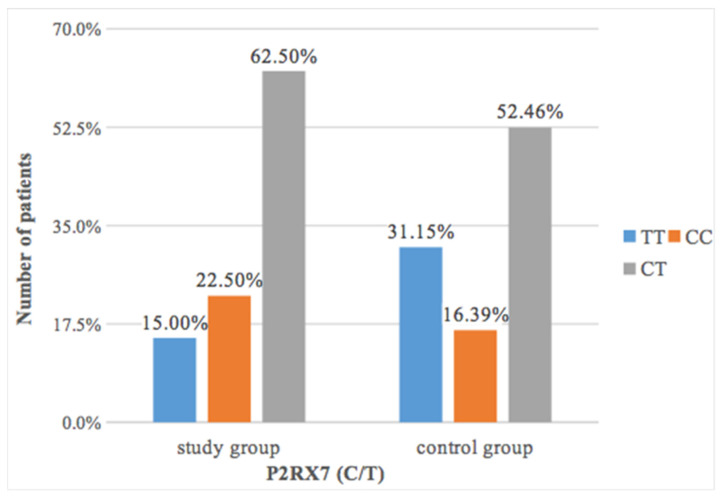
The prevalence of *P2RX7* polymorphism in the study and control groups.

Statistical analysis showed that patients with periapical resorption had a slightly more frequent CC polymorphism of the *P2RX7* gene (22.50%) than patients in the control group (16.39%) (Figure 1).

The differences found were not statistically significant (*p* = 0.17) (Table 5).

Statistical analysis showed a significant relationship between the occurrence of *P2RX7* polymorphism (C/T) and the Malmgren and Levander index (Table 6). Grades 3 or 4 occurred more than twice as often in the case of CC (6.84%) and almost twice as often in the case of CT (5.61%) than in the case of TT (3.20%). Grade 0 occurred most frequently in the case of TT (18.80%), much less frequently in the case of CT (11.75%), and definitely the least often in the case of CC (6.84%).

**Figure 2:**
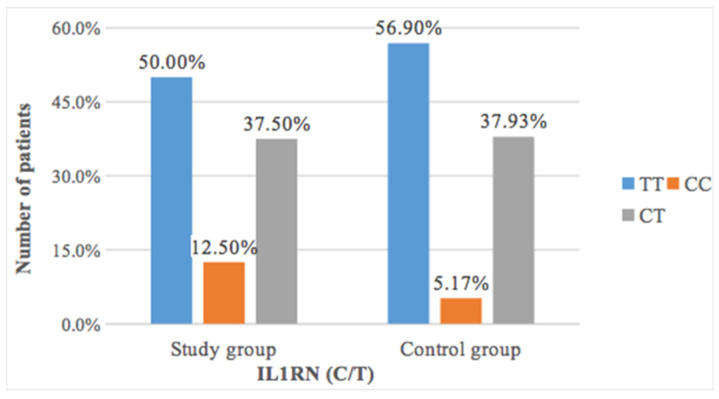
The prevalence of *IL1RN* polymorphism in the study and control group.

The study of the occurrence of thymine to cytosine transduction revealed a more frequent occurrence of CC polymorphism in patients with resorption after orthodontic treatment (12.5%) than in patients without changes in root length (5.17%) (Table 7 and Figure 2). However, this difference was not statistically significant (*p* = 0.41).

Statistical analysis showed a significant relationship between the occurrence of *IL1RN* (C/T) polymorphism and the Malmgren and Levander classification. Grades 3 or 4 occurred more than twice as rarely in the case of TT (4.15%) and almost twice as rarely in the case of CT (5.61%) than in the case of CC (10.0%). In the case of CC polymorphism, one may also notice twice as frequent occurrence of grade 2 and more than twice as rare occurrence of grade 1 than in the case of other polymorphisms (Table 8).

## 3. Discussion

EARR is a frequent iatrogenic result of orthodontic treatment, especially in the maxillary incisors, due to compression of periodontal ligaments. This compression causes reduction or interruption of microcirculation; it may result in sterile necrosis. During reduction of this necrotic tissue by microphages, root integrity may be damaged [30]. As was widely observed in the studies published earlier, the most affected teeth included maxillary incisors [31,32]. Also in the conducted study, resorption was the most frequently present in maxillary incisors, of which the most advanced resorption concerned the maxillary right lateral incisor. In the studies of other authors, only 5% of all the examined teeth indicated greater EARR than 20% of root length reduction, which is consistent with the present study, in which resorptions with the loss over 20% of root length amounted to 5.75% [33].

In the search for genetic susceptibility, association studies have proposed several polymorphisms of genes involved in bone metabolism and root remodeling. However, all of the studies are of small sample size compared to most association studies, which still tend to find a variable amount of the total variation to be accounted for by specific genetic variants. Our data may be useful in future meta-analyses to increase the sample size and power to determine effect size, albeit in groups and not individuals.

The post-orthodontic EARR has been the subject of much recent research. Pereira et al. demonstrated the influence of both clinical and genetic parameters, i.e., the GG variant in the *P2RX7* gene (rs1718119) on the development of EARR [34]. Sharab et al. reported that two of the tested SNPs (+1068A/G, rs1718119, Thr348Ala, and +1405A/G, rs2230912, Gln460Arg) occurred with a similar frequency in the study group and in the control group, while the patients with the third SNP (+489C/T, rs208294, Tyr155His) were more prone to resorption [35]. Long treatment length and the presence of the CC and CT genotypes for *P2RX7* SNP were significantly associated with EARR in their study [35].

In Czech patients, the CG variant of the *P2RX7* gene was more frequent in the study group than in the control group [28].

*P2RX7* is a gene that codifies the purinergic receptor P2X ligand-gated ion channel 7. In the polymorphism studied, T-thymine is converted to C-cytosine in the TAT codon encoding tyrosine (Tyr). As a result of the mutation, an amino acid is replaced, tyrosine (Tyr) is replaced by histidine (His). This is a missense mutation that can result in changes in the organism’s phenotype. This is one of the possible mechanisms explaining the increased incidence of EARR in patients with the CT variant in the *P2RX7* gene. Cabrini et al. proved that polymorphism 489C > T (H155Y) enhancing the function of the receptor, increases Ca ions influx in the HEK293 cells line (recombinant HEK293 cells expressing P2X7R) and lymphocytes of patients with chronic lymphocytic leukemia (CLL) [36]. Those authors observed that the change of electrostatic charges consequent to His to Tyr substitution and the potential of Tyr phosphorylation could facilitate the recruitment of the P2X7 monomers in the process of assembly of the receptor complexes or a stabilization of the active complexes themselves, thus increasing the ratio between active complexes and inactive monomers. Calcium ions are associated with all phases of apoptosis: as a relay in the initial phase, as the ion inducing the mitochondrial mega-channel during the effector phase, and as an activator of transglutaminases, proteases and endonuclease active in the degradation phase [37]. In our polymorphism allele, T function may be connected with the change of receptor conformation to enhance the function of P2X7R receptor as a membrane channel and therefore, increase Ca ions influx to cytoplasm in vitro in the HEK293 cell line, as well as in circulating blood lymphocytes PBL of patients with CLL. Additionally, enhancing the function of receptor acts pro-inflammatorily by activating interleukin-1β.

In our own studies it was proved that in patients with apical resorptions slightly more often occurred CC polymorphism in the *P2RX7* (rs208294) (22.50%) than in patients from the control group (16.39%), although those differences were not statistically significant. Statistical analysis also found a significant relationship between the occurrence of polymorphism *P2RX7* (C/T) and Lavender and Malmgren’s index. The advanced grades 3 or 4 resorption was present over twice more often in the case of CC and almost twice more often in the case of CT than in the case of TT. A 0 grade most often occurred in the case of TT (18.80%), significantly less frequently in the case of CT (11.75%), while the least often in the case of CC (6.84%).

Some researchers concluded that polymorphism in *IL1RN* may be correlated with EARR [29,38]. Iglesias-Linares et al. proved that TT *IL1RN* SNP rs419598 genotype is strongly connected with EARR, however in the study by Guo et al., no statistically significant relationship between EARR and SNP *IL1RN* was found [29,38]. Nevertheless, it was noticed that despite lack of statistically significant difference, average volume of root resorption in patients with TT variant was still greater than in patients with CT genotype. This result could be an effect of limited sample size. In our own studies an analysis of the occurrence of thymine to cytosine transition showed the occurrence of CC polymorphism in patients with resorptions after the orthodontic treatment than in patients without resorption, on the contrary to the previous researchers. However, this difference was not statistically significant (*p* = 0.41). Statistical analysis also found a significant relationship between the occurrence of *IL1RN* (C/T) polymorphism and Lavender and Malmgren’s classification. The grades 3 or 4 occurred more than twice less frequently in the case of TT (4.15%) and almost twice less often in the case of CT (5.61%) than in the case of CC (10.0%), which suggests that CC variant increased the risk of resorption in the studied sample.

There were 4 comparisons in our study and, therefore, without applying the adjustment procedures, the error I would be 1 − (1 − 0.05)^4^ ≈ 0.185 (18.5%). A Bonferroni correction has been introduced to avoid increasing the type I error. To maintain the probability of at least one type I error at the level of 5%, each test must be performed at a significance level of 0.05 / 4 = 0.0125. Then, the probability of making one type I mistake is: 1 − (1 − 0.05/4)^4^ ≈ 0.0491, which means less than 5%. The entire study retains a significance level of 0.05. Thus, significance and dependence at the level of α = 0.05 was found at *p* < 0.0125. This does not change anything in our conclusions, because both statistically significant results are below 0.0125 (Table 8: *p* = 0.000051 and Table 6: *p* = 0.001625).

The association of SNPs in selected genes with postorthodontic EARR in this study should be regarded with caution due to the small pool of patients included in the study, despite the fact that the number of samples obtained was comparable to other studies. Another limitation of the study were the two-dimensional X-rays (orthopantomograms and cephalograms) used to assess the degree of resorption. The lack of an indication for CBCT imaging justifies the use of routine diagnostic pantomograms and cephalograms, taking into account the fact that the tests showed similar sensitivity of both methods [39,40].

## 4. Conclusions

In conclusion, our data suggest that the variability in the *P2RX7* and *IL1RN* genes may be the risk factors predisposing to the development of postorthodontic EARR. However, EARR is a complex disease with a multifactorial etiopathogenesis, therefore each SNP can only partially affect the observed clinical phenotype.

Due to the small number of patients included in this analysis, the number of comparisons performed, and only marginally significant differences, more comprehensive studies on variations in the *IL1RN* gene and *P2RX7* gene in other populations could help define the true role of these markers as a risk factor for the development of postorthodontic root resorption.

## 5. Material and Methods

### 5.1. Subjects

The study involved 101 (77 females, 24 males) Polish patients treated in a private clinic ORTO-PUNKT in Tarnów. The mean age of the patients was 22 years/9 months (±6 years/3 months). The mean age of the female patients was 21.08 years (±7.32 years). The mean age of the male patients was 22.08 years (±7.23 years). The average duration of orthodontic treatment was 31.1 months (±6.4 months). On the basis of the cephalometric analysis, 53 patients were qualified to the skeletal class I, 41 patients to the skeletal class II, and 7 patients to the skeletal class III. In 8 patients with class II malocclusion, extraction was performed in order to camouflage the defect. In almost half of patients (42.57%) before setting up a fixed appliance, an additional appliance for expansion or dystalisation was used. All the patients underwent a complex orthodontic treatment using fixed braces (straight arch technique). They had to meet the following criteria: complete anamnestical and clinical data available, clearly assessable X-ray radiographs (orthopantomograms and lateral cephalometric radiographs) taken before and at the end of the treatment using the same machine, examined teeth free of fractures or abrasion on the incisal edges between measurements. Patients with dental trauma, incomplete root development, or previous orthodontic treatment with a fixed appliance were excluded from the study. In addition, people with systemic disease affecting dental hard tissues were also excluded from the examination. Patients were not related to each other. All the patients were treated by one doctor.

All the subjects gave their informed consent for inclusion before they participated in the study. The study was conducted in accordance with the Declaration of Helsinki, and the protocol was approved by the Ethics Committee of Medical University of Lublin, Poland (KE-0254/335/2018, approval date 20 December 2018).

### 5.2. Radiographic Measurements

**Figure 3:**
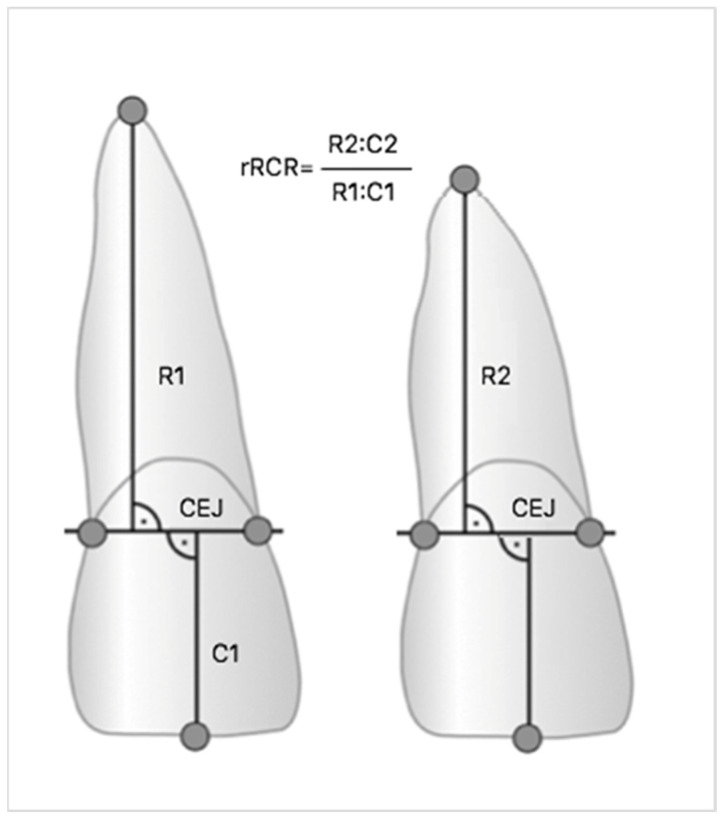
Measurement of the panoramic radiographs (CEJ = cemento-enamel junction): root and crown lengths assessed to determine individual root-crown-ratio (RCR).

Incisors of the maxilla and mandible as well as upper first molars were selected for measurement. Root resorption already occurs during the initial phase of therapy, the most affected teeth being the incisors [41,42,43,44]. Roots were measured based on orthopantomograms taken before and at the end of treatment, as in the previous studies [17,18,28,35,45]. Measurements were made on digital radiographs using diagnostic software (Planmeca Romexis Viewer), which allowed the highest precision when locating root endpoints. All images have been previously calibrated. Based on studies by Linhartova and Pereira, the Linge and Linge method modified by Brezniak et al. was chosen, and both individual root-crown ratio (RCR) and the relative changes of RCR (rRCR) were calculated [28,34,46,47]. The ratio of crown length before and after the treatment (C1/C2) was used as a C1/C2 strengthening factor, because it is assumed that during orthodontic treatment the length of the crown does not change [48]. Therefore, an rRCR of 100% indicates no change of root length after treatment relative to the pre-treatment root length. If during the procedure the root became shorter, the degree of root resorption was calculated based on the equation R1-R2 (C1/C2) and the rRCR decrease (Figure 3). Next, due to concern about the accuracy of the Linge and Linge method demonstrated by Katona, the Malmgren and Levander’s method was used to assess the root resorption [49,50]. All subjects were divided into two groups based on the presence or absence of postorthodontic EARR resulting from radiographic measurements: a control group of 61 patients without EARR (with 0.90 ≤ rRCR ≤ 1.00) and a study group of 40 patients with EARR (rRCR < 0.90).

### 5.3. Reliability of the Method

Reproducibility of measurements was statistically evaluated by comparing double measurements on X-rays of 10 randomly selected patients. The interval between the respective measurements was 3 weeks. The method error was calculated based on the Dahlberg formula (S = √Σd2/2n), where d is the difference between the repeated measurements and n is the number of repeated measurements with pairs [51]. The mean error for the tooth length measurements was 0.33 mm.

### 5.4. Sample Collection and Determination of the P2RX7 and IL1RN Genotypes

The research material was a swab from oral cavity collected from 101 patients using a disposable, sterile stub of swab stick with transport medium. Each stub of swab stick containing patient material was placed in a sterile Eppendorf tube. Then, 500 µL Lysis buffer was added to each Eppendorf tube and shaken on a vortex (2500 rpm for 1 min), then the samples were placed in the refrigerator for about 30 min, stirring every 5 min. After this time, a stub of swab stick was removed and discarded from each tube and the tubes were sealed.

Isolation of DNA from cell suspension was obtained by the Hirt method (two days method). Samples with isolated DNA were placed in a centrifuge cooled to 4 °C, centrifuged for 15 min at 13.2 thousand rotations per minute. After this time, ethyl alcohol was removed from each sample. The samples were then left opened for approximately 5 min at room temperature until the ethanol evaporated completely. After ensuring that the samples were ethanol-free, 22 mL of ultra pure nuclease-free water was added to each to dissolve the DNA, and then the samples were placed on ice. The concentration of isolated DNA was measured on a NanoDrop P2000C spectrophotometer. Initially, before measuring DNA concentration, the device was calibrated. An amount of 2 μL of ultra pure nuclease-free calibrator water was transferred to the spectrophotometer window. Samples were measured by applying 2 µL of patient DNA to the measurement window. DNA concentration was determined at 260 nm. The assessment of the degree of purity of isolated nucleic acid (DNA) is the factor (A260/A280)—the absorbance ratio at 260 nm to 280 nm. The purity of the isolated DNA was in the range 1.8–2.05, so the samples were taken for further analysis.

Polymorphism of two genes—*P2RX7* (rs208294) and *IL1RN* (rs419598)—was studied. Each probe was diluted to a concentration of 20× with TE buffer, and then reaction mixtures were prepared—for each probe separately. The reaction mixture was prepared from 12.50 µL TaqMan Genotyping PCR Master Mix and 1.25 µL previously adjusted to 20x probe concentration (SNP Genotyping Assay). SNP analysis was performed on a 96-well plate using real-time PCR on a StepOnePlus System instrument. For each probe, the reaction was carried out on a separate plate. An amount of 11.25 µL of sample (DNA-H2O) and 13.75 µL of reaction mixture were added to each well. Two negative controls (NTC) were performed on each plate. Nuclease-free water and the appropriate reaction mixture were used as negative controls. The reaction proceeded under different conditions depending on the polymorphism studied.

### 5.5. Statistics

The results of genetic examination and the comparison of X-ray images were subjected to statistical analysis. The statistical analysis used the verification of statistical hypotheses based on Pearson’s Chi2 test. This test was used to study the relationship between the qualitative data.

The study results were prepared using the “STATISTICA 13.3” program and Microsoft Excel 2016. The significance of differences and relationships was found at a *p*-value < 0.05. Due to the use of multiple tests, a Bonferroni correction was introduced to avoid increasing the type I error.

## Figures and Tables

**Table 1:** Root resorption of the studied teeth.

rRCR (Relative Changes of Root-Crown Ratio)	Number (n)	Percentage (%)
rRCR < 0.80	23	2.28
0.80 ≤ rRCR < 0.90	70	6.93
0.90 ≤ rRCR < 1.00	898	88.91
Total	1010	100.0

**Table 2:** Root resorption of teeth in the study group.

rRCR	Number (n)	Percentage (%)
rRCR < 0.80	23	5.75
0.80 ≤ rRCR < 0.90	69	17.25
0.90 ≤ rRCR < 1.00	301	75.25

**Table 3:** Root resorption of teeth in the control group.

rRCR	Number (n)	Percent (%)
rRCR < 0.80	0	0
0.80 ≤ rRCR < 0.90	0	0
0.90 ≤ rRCR < 1.00	598	98.03
Total	610	100.0

**Table 4:** Distribution of resorption of particular teeth in total.

rRCR		Tooth									
		11	12	16	21	22	26	31	32	41	42
rRCR < 0.80	n	2	5	0	2	3	0	4	1	3	3
	%	1.98	4.95	0.00	1.98	2.97	0.00	3.96	0.99	2.97	2.97
0.80 ≤ rRCR < 0.90	n	6	13	1	6	9	1	10	9	8	7
	%	5.94	12.87	0.99	5.94	8.91	0.99	9.90	8.91	7.92	6.31
0.90 ≤ rRCR < 1.00	n	91	82	97	91	87	98	86	88	89	89
	%	90.10	81.19	96.04	90.10	86.14	97.03	85.15	87.13	88.12	88.12
rRCR ≥ 1.00	n	2	1	3	2	2	2	1	3	1	2
Total	n	101	101	101	101	101	101	101	101	101	101
	%	100.0	100.0	100.0	100.0	100.0	100.0	100.0	100.0	100.0	100.0

**Table 5:** The occurrence of the *P2RX7* polymorphism in the study and control groups.

*P2RX7* (C/T)	Group	
	Study	Control
**TT**	6	19
	15.00%	31.15%
**CC**	9	10
	22.50%	16.39%
**CT**	25	32
	62.50%	52.46%
**Total**	40	61
	100.0%	100.0%

Chi2 Pearson’s: 3.455321; *p* = 0.17770.

**Table 6:** Occurrence of *P2RX7* genotypes according to Malmgren and Levander index.

Malmgren and Levander Index	*P2RX7* (C/T)		
	TT	CC	CT
**0**	47	13	67
	18.80%	6.84%	11.75%
**1**	100	86	278
	40.00%	45.26%	48.77%
**2**	95	78	193
	38.00%	41.05%	33.86%
**3 or 4**	8	13	32
	3.20%	6.84%	5.61%
**Total**	250	190	570

Chi2 Pearson’s: 21.2930; *p* = 0.001625.

**Table 7:** The prevalence of *IL1RN* (C/T) polymorphism in the study and control group.

*IL1RN* (C/T)	Group	
	Study	Control
**TT**	20	33
	50.00%	56.90%
**CC**	5	3
	12.50%	5.17%
**CT**	15	22
	37.50%	37.93%
**Total**	40	58
	100.0%	100.0%

Chi2 Pearson’s: 1.766475; *p* = 0.41344.

**Table 8:** Occurrence of *IL1RN* genotypes according to Malmgren and Levander index.

Malmgren and Levander Index	*IL1RN* (C/T)		
	CT	CC	CT
**0**	60	12	55
	11.32%	15.00%	14.86%
**1**	259	16	171
	48.87%	20.00%	46.22%
**2**	189	44	121
	35.66%	55.00%	32.70%
**3 or 4**	22	8	23
	4.15%	10.00%	6.22%
**Total**	530	80	370
	100.0%	100.0%	100.0%

Chi2 Pearson’s: 29.4211; *p* = 0.000051.

## Data Availability

The data presented in this study are available on request from the corresponding author. The data are not publicly available due to privacy.

## Abbreviations

SNP	Single Nucleotide Polymorphism
*IL1RN*	Interleukin 1 Receptor
*P2RX7*	Purinoreceptor P2 × 7
EARR	External Apical Root Resorption
rRCR	Relative changes of Root-Crown Ratio
ATP	Adenosine Triphosphate
IL-1b	Interleukin 1 beta
OPG	Osteoprotegerin
RANKL	Receptor Activator Nuclear Factor Kappa Ligand
RANK	Receptor Activator Nuclear Factor Kappa
CLL	Chronic Lymphocytic Leukemia
CBCT	Cone Beam Computed Tomography
